# Nonclinical safety assessment of repeated administration and biodistribution of ChAd3‐EBO‐Z Ebola candidate vaccine

**DOI:** 10.1002/jat.3941

**Published:** 2020-01-21

**Authors:** Camille Planty, Guillaume Chevalier, Marie‐Ève Duclos, Clémentine Chalmey, Catherine Thirion‐Delalande, Cécile Sobry, Ann‐Muriel Steff, Eric Destexhe

**Affiliations:** ^1^ GSK, Rixensart Belgium; ^2^ Citoxlab Évreux France; ^3^ GSK Rockville Maryland USA

**Keywords:** adenovirus vector, biodistribution, ChAd3, Ebola virus, toxicity, vaccine

## Abstract

ChAd3‐EBO‐Z is an investigational adenovirus‐based vaccine for the prevention of Ebola virus disease. Two nonclinical studies were performed to evaluate the biodistribution, local tolerance and potential local and systemic toxic effects of this vaccine. In the biodistribution study, rats received a single intramuscular injection of either ChAd3‐EBO‐Z or saline. Enlargement of the draining lymph nodes, starting on day 2, was noticed in ChAd3‐EBO‐Z‐treated rats, indicating that an immune response had taken place. Viral DNA was mainly found at the injection sites and in the draining lymph nodes, from where it progressively disappeared during the observation period, while it was found only transiently and occasionally in other organs. In the repeated‐dose toxicity study, either ChAd3‐EBO‐Z or saline was administered intramuscularly to rabbits on two occasions with a 2‐week interval. General health status, rectal temperature, local tolerance, ophthalmology, hematology, coagulation and blood chemistry parameters were monitored. Macroscopic and microscopic evaluations were performed. Treatment‐related changes included a transient increase in neutrophil count, C‐reactive protein and fibrinogen levels, and a transient decrease in platelet count. As expected, microscopic observations 3 days after the second injection were related to the elicited inflammatory reaction, and these inflammatory responses had almost completely disappeared 29 days after the second immunization. In conclusion, the vaccine was locally and systemically well‐tolerated and the viral vector was partially or totally cleared from the organs where it disseminated, supporting the clinical development of the vaccine.

## Abstract 2

ChAd3‐EBO‐Z is an investigational vaccine for the prevention of Ebola virus disease. The local tolerance and the potential local and systemic effects of this vaccine were evaluated in rabbits and its biodistribution in rats. The vaccine was locally and systemically well‐tolerated and the viral vector was partially or totally cleared from the few organs where it disseminated, thus supporting the clinical development of the vaccine.

## 1 INTRODUCTION

Ebola virus disease (EVD), or Ebola hemorrhagic fever, is caused by the Ebola virus (EV) of the *Filoviridae* family. EVD is characterized by a sudden onset of fever and weakness, accompanied by other symptoms such as headache, muscle pain, conjunctivitis, rash, and diarrhea and vomiting, with a proportion of individuals presenting hemorrhagic symptoms. EV directly causes tissue damage, either by direct cytopathic effects or through indirect effects related to the release of inflammatory mediators or alteration of vascular functions. This results in coagulation disorders and the destruction of several organs, including the liver and kidneys (Feldmann & Geisbert, 2011). Starting in late 2013 up to 2016, the world's largest EVD outbreak was recorded in West Africa (Coltart, Lindsey, Ghinai, Johnson, & Heymann, 2017). This epidemic, caused by the Zaire species (one of the six known *Ebolavirus* species), resulted in more than 11 000 deaths (WHO, 2016), which prompted different groups to react quickly and accelerate Ebola vaccine development (Lambe, Bowyer, & Ewer, 2017).

In this context, an investigational vaccine for EVD prevention, ChAd3‐EBO‐Z, composed of the replication‐defective chimpanzee adenovirus 3 vector (ChAd3) with a DNA fragment encoding the Ebola Zaire glycoprotein (GP) was developed. It showed encouraging nonclinical efficacy, inducing protection against acute lethal EV challenge in nonhuman primates (Stanley et al., 2014).

To support the development of the ChAd3‐EBO‐Z vaccine, a number of nonclinical toxicity studies were performed. This article reports two studies performed in rats and rabbits under Good Laboratory Practice (GLP) principles. The objectives were to evaluate the biodistribution of ChAd3‐EBO‐Z after a single intramuscular (IM) injection in rats, and the local tolerance, potential local and/or systemic toxic effects, and persistence, delayed onset or reversibility of any effects in rabbits after two IM injections.

## 2 MATERIALS AND METHODS

### 2.1 Animals

Male and female Sprague‐Dawley rats were obtained from Janvier Labs and acclimated to the study conditions for 7 days before the beginning of the study. On the first day of treatment, the animals were 7 weeks old. The males weighed 280‐314 g and the females weighed 192‐229 g. They were kept in polycarbonate cages (n = 2‐3 animals; same sex and treatment group) containing autoclaved sawdust. Treated and control animals were housed in separate dedicated rooms each with filtered air (8‐15 air changes per hour) at a temperature within the range 20‐24 °C and relative humidity within the range 30%‐70%. The lighting followed a 12‐hour light/12‐hour dark cycle. Rats had free access to a standard laboratory rat diet (SSNIFF R/M‐H; SSNIFF Spezialdiäten GmbH) and had free access to 0.22‐μm filtered drinking water. Each cage contained a rat hut for environmental enrichment.

SPF‐bred male and female New Zealand White albino rabbits were obtained from Centre Lago (Vonnas, France), and were acclimated to the study conditions for 14 days. On the first day of treatment, the animals were 4‐5 months old. The males weighed 3100‐3800 g and the females weighed 3300‐4000 g. They were individually housed in polycarbonate cages over trays, in dedicated rooms with filtered air (5‐15 air changes per hour), at a mean temperature within the range 15‐21 °C and relative humidity within the range 30%‐70%. The lighting followed a 16‐hour light/8‐hour dark cycle. The rabbits were provided ad libitum with standard laboratory rabbit diet (Type 110 C; SAFE) and had free access to 0.22‐μm filtered tap water. Dumbbells were placed in the cages for environmental enrichment.

The welfare of the animals was maintained in accordance with the General Principles Governing the Use of Animals in Experiments (Directive 2010/63/EU). The Citoxlab France Ethics Committee reviewed both study plans to assess compliance with the Directive 2010/63/EU. The CEC notified the Study Directors of the review before finalization of the study plans.

### 2.2 Control and test items

The control item was a ready‐to‐use nonpyrogenic, sterile 0.9% saline formulation. The ChAd3‐EBO‐Z was provided frozen in monodose vials containing 1.2 mL solution at 0.8 × 10^11^ viral particles/mL (Vp/mL) and was kept at −80 °C until use. ChAd3‐EBO‐Z consists of a recombinant replication‐deficient adenovirus chimpanzee serotype 3 vector expressing wild‐type EV from the Zaire Mayinga species (Volchkov et al., 1997).

### 2.3 Description of the studies

Throughout the text, Study 1 is the biodistribution study in rats, and Study 2 is the repeated‐dose toxicity study in rabbits. Both studies were carried out at Citoxlab Laboratories (Évreux, France). They were conducted in compliance with Directive 2004/10/EC of the European Parliament and the Council of 11 February 2004 on the harmonization of laws, regulations and administrative provisions relating to the application of the GLP principles (February 2004).

Both study designs were based on the following guidelines: EMEA CPMP/SWP/465/95 December 1997, Note for guidance on the preclinical pharmacological and toxicological testing of vaccines; WHO guidelines on the nonclinical evaluation of vaccines, Technical Report series no. 927, 2005; and WHO guidelines on the nonclinical evaluation of vaccine adjuvants and adjuvanted vaccines (2013).

In addition, Study 1 followed the European Medicines Agency (EMA) CHMP/VWP/141697/2009 Guideline on quality, nonclinical and clinical aspects of live recombinant viral vectored vaccine; the EMEA/273974/2005 Guideline on nonclinical testing for inadvertent germline transmission of gene transfer vectors; and the CBER/OCTGT Guidance for Industry (2013), preclinical assessment of investigational cellular and gene therapy products, and Study 2 followed the Guideline on repeated‐dose toxicity, Committee for Human Medicinal Products (CPMP/SWP/1042/99 Rev 1), EMA, 18 March 2010; the Guideline on adjuvants in vaccines for human use (CHMP/VEG/134716/2004); and the Note for guidance on nonclinical local tolerance testing of medicinal products (CPMP/SWP/2145/00).

#### 2.3.1 Study 1: biodistribution after a single injection in rats

Rats in the treatment group were randomly allocated to four subgroups (one for each time point) of 10 animals, each composed of five males and five females. These animals received one IM injection (200 μL) of the test item ChAd3‐EBO‐Z (1.6 × 10^10^ Vp/animal) in the right quadriceps. This represents approximately 1/10th of a human dose, meaning 20‐ and 30‐fold the human dose relative to body weight for males and females, respectively. Treated animals were necropsied 24 hours or 7, 28 or 48 days after administration (i.e., days 2, 8, 29 and 49, respectively). These time points were determined based on data generated in a former biodistribution study performed with another adenovector (unpublished data). In the control group, four males and four females received an injection of saline solution, and one animal/sex was necropsied at the same time point as in the treated group.

##### Clinical examinations

The animals were checked daily for mortality and clinical signs of illness. Food consumption was recorded at least once a week and was calculated per animal and per day based on group average. The body weight of each animal was recorded before treatment, on the day of treatment and then at least once a week (or on day 2 for animals necropsied 24 hours after treatment).

##### Pathology

On completion of the observation period, the animals were killed and a full macroscopic post‐mortem examination was performed. Designated organs were weighed and selected tissue specimens were preserved for ChAd3‐EBO‐Z tissue distribution analysis by quantitative polymerase chain reaction (qPCR), as described below.

##### Evaluation of biodistribution by quantitative polymerase chain reaction

Blood was collected before necropsy from the abdominal aorta or vena cava of animals (under deep anesthesia) into potassium EDTA tubes. Blood was aliquoted, snap frozen in liquid nitrogen and then stored at −20 °C until DNA extraction.

Tissue samples were collected in the following order: brain, lung, liver, spleen, kidneys, heart, gonads, lymph nodes (popliteal, inguinal and iliac), and muscle at the injection site. A clean set of disposable instruments was used for each organ. Samples were rinsed with phosphate‐buffered saline without calcium and magnesium. Adipose tissue was carefully removed from the organs. For large organs, three samples weighing approximately 100 mg were taken. Small organs were sampled as a whole. Tissue samples were snap frozen in liquid nitrogen and stored at −20 °C until DNA extraction.

DNA was first extracted from blood and tissue samples collected on days 2 and 8, and then DNA was extracted from samples collected on day 29 when the corresponding tissues were positive on day 2 and/or 8. Likewise, DNA was extracted from samples collected on day 49 only if the corresponding blood or tissue samples were positive on day 29.

DNA was extracted from blood using the Blood Quick Pure kit (Macherey Nagel) and from tissue samples using the NucleoSpin tissue kit (Macherey Nagel), according to the manufacturer's instructions. Briefly, tissue samples were first incubated in a proteinase K solution before DNA extraction. Chaotropic salts and ethanol were added to the lysate to obtain appropriate conditions for DNA binding to the silica membrane. After washing, DNA was eluted under low ionic strength conditions in a slightly alkaline elution buffer. Tissues from control animals were extracted first.

Extracted DNA was used as template for PCR amplification with a validated qPCR test carried out on an ABI 7900 HT Fast Real‐Time PCR system (Life Technologies).

The sequence of the forward primer (targeting the CMV promoter) was 5′‐ATCTACGTATTAGTCATCGCTATTACCA‐3′; the sequence of the reverse primer was 5′‐GACTTGGAAATCCCCGTGAGT‐3′, and the sequence of the internal TaqMan probe was 6FAM‐5′‐ACATCAATGGGCGTGGATAGCGGTT‐3′‐TAMRA. DNA (0.4 or 0.2 μg for lymph nodes and blood) in elution buffer was amplified in a mixture containing TaqMan Universal master mix, DNase/RNase‐free water, both primers and the dual hybridization probe. Amplification was detected in real‐time over 40 cycles (including an elongation step at 60 °C) by following the evolution of the fluorescent signal generated by the degradation of the probe. Samples were analyzed in duplicate, in parallel with a calibration curve performed with known amounts of ChAd3‐EBO‐Z. The *C*_t_ values were plotted against the logarithm of the Vp numbers of calibration points and the resulting curve was linearized. For all samples, test item Vp numbers were calculated by interpolation from the calibration curve and adjusted by a dilution factor to express the results in Vp number/μg DNA. The upper limit of quantification was 10^7^ Vp/well (corresponding to 2.5 × 10^7^ Vp/μg DNA when 0.4 μg DNA was assayed), the lower limit of quantification was 100 Vp/well (corresponding to 250 Vp/μg DNA) and the limit of detection was 25 Vp/well (corresponding to 62.5 Vp/μg DNA).

DNA extracted from biological samples may contain PCR inhibitors, leading to artificial underestimation of the results. Thus, PCR inhibition was checked in all tested samples by spiking a known quantity of exogenous DNA in the samples and measuring this DNA. Inhibition was revealed by discrepancy between expected and measured amounts of exogenous DNA.

#### 2.3.2 Study 2: Repeated dose toxicity study in rabbits

The rabbit was chosen for the toxicity evaluation based on recommendations by EMA and WHO for this type of investigation (EMA‐CPMP, 1997; WHO, 2003), and on previous experience. The relevance of this species was demonstrated through a serological analysis conducted before the initiation of the study (data not shown).

The dosing schedule was intended to cover the number of injections foreseen in humans plus one extra. Rabbits were randomly allocated into two groups of 20 animals, each composed of 10 males and 10 females. One group received 2 mL IM injections of ChAd3‐EBO‐Z (1.6 × 10^11^ Vp/animal, corresponding to the full human dose) on days 1 and 15. The vaccine was administered as two 1‐mL injections. On day 1, one injection was made in the anterior right thigh muscle (injection site 1) and the other in the anterior left thigh muscle (injection site 2). On day 15, one injection was made in the posterior right thigh muscle (injection site 3) and the other in the posterior left thigh muscle (injection site 4). The control group received IM injections of saline. Five animals/sex/group were killed on day 18 (early necropsy, 3 days after the second injection), and the remaining five animals/sex/group were killed on day 43 after a 29‐day treatment‐free period (late necropsy, 29 days after the second injection).

##### Clinical examinations

The animals were checked twice a day throughout the study for mortality and clinical signs of illness. Local reactions at the injection sites were recorded 3, 24, 48 and 72 hours after each injection. The body weight of each animal was recorded once before group allocation (day −12), before the first injection (day −5), daily for 1 week following the first injection, on day 10 and before the second injection (day 15), for three consecutive days following the last injection (days 16, 17, 18), then on days 25, 30, 35, 42 and 43. The quantity of food consumed by the animals was recorded twice 4 days before the beginning of the treatment period (on days −4 and −2) and on days 1, 3, 5, 7, 9, 11, 13, 14, 17,19, 21, 23, 25, 27, 29, 31, 33, 35, 37, 39, 41 and 43 during the treatment and treatment‐free periods. Food consumption was calculated per animal and per day. Rectal temperature was measured in late necropsy animals, once before each injection, and 3, 8, 24 and 48 hours after each administration and then once the day before being killed (day 42). Ophthalmological examinations were performed on late necropsy animals before the beginning of the treatment period and on days 18 and 42.

##### Blood sampling, clinical chemistry and hematology

Hematology, coagulation and blood biochemistry investigations were performed for each animal, once before the beginning of the treatment period, 8 hours after the first injection (day 1), on days 2 (hematology parameters only), 3, 8 and 12, just before the second injection (day 15), 8 hours after the second injection (day 15), and on days 16, 17 and 18 (for the last two hematology parameters only). In addition, blood samples were also taken on days 22 and 42 from late necropsy animals only.

For hematology parameters, blood was collected into potassium EDTA tubes and the blood composition was analyzed using the ADVIA 120 Hematology Analyzer. Blood smears were microscopically examined if the blood sample was not accepted by the analyzer. Microscopic evaluation was also systematically performed for the identification and gradation of platelet aggregates when platelet count was <100 G/L. Blood was also collected into citrate tubes and analyzed using ACL Elite Pro blood coagulation analyzer for the determination of prothrombin time, fibrinogen level and activated partial thromboplastin time (APTT).

For clinical chemistry analyses, blood was taken into heparinized tubes and analyzed using the ADVIA 1650 or 1800 blood biochemistry analyzer. The parameters measured were alkaline phosphatase, alanine aminotransferase, aspartate aminotransferase, creatine kinase, glucose, total protein, albumin (and the albumin/globulin ratio), urea, creatinine, bilirubin, cholesterol, triglycerides, calcium, sodium, potassium, chloride and inorganic phosphate. C‐reactive protein (CRP) levels were determined in each animal, once before the beginning of the treatment period, then on days 3, 8, 12 and 16, and, for late necropsy animals only, on days 22 and 42.

##### Serology

Blood samples for the determination of anti‐GP antibodies (immunogenicity, non‐GLP) were taken from all animals before the beginning of the treatment period and on days 18 (all animals) and 42 (late necropsy animals). A noncommercial enzyme‐linked immunosorbent assay (ELISA) was used to quantify GP‐specific antibodies in rabbit serum to evaluate vaccine uptake. The animals were confirmed as being exposed to the vaccine when anti‐GP IgG geometric mean titers (GMT) >23.8 μg/mL.

##### Necropsy, tissue processing and histopathological examination

The rabbits were deeply anesthetized by an intravenous injection of sodium pentobarbital and then euthanized by exsanguination. They were examined for external changes and gross pathological changes. Special attention was paid to the macroscopic examination of the injection sites.

Adrenals, brain, lymph nodes, epididymides, heart, kidneys, liver, ovaries (females), pituitary gland, prostate (males), spleen, testes (males), thymus, thyroid glands with parathyroid glands and uterus (females) were weighed and subsequently preserved in phosphate‐buffered neutral 10% formaldehyde for microscopic evaluation. Other organs were not weighed but were preserved for examination, including aorta, colon, eyes and optic nerves (fixed in modified Davidson's fixative), femoral bone, gall bladder, gut‐associated lymphoid tissue, larynx, lungs with bronchi, pancreas, salivary glands, sciatic nerve, triceps muscle, spinal cord, sternum with bone marrow, stomach, urinary bladder and vagina (females). All tissues submitted for histopathological evaluation were embedded in paraffin wax, sectioned at 4 μm and stained with hematoxylin and eosin.

To analyze the injection sites, the complete muscles were collected and preserved in phosphate‐buffered neutral 10% formaldehyde. Three approximately 5‐mm pieces from around the injection site were prepared, examined macroscopically for gross findings and processed for microscopic evaluation in three semi‐serial sections, 150 μm apart, for each piece. Peer review was performed on 60% of the animals from the test item treatment group, and on an adequate number of slides from identified target organs to confirm that findings recorded by the study pathologist were consistent and accurate.

Finally, bone marrow smears were prepared from the sternum of each animal.

### 2.4 Statistical analysis

For the comparison of mean body weight, mean body temperature, hematology, coagulation or clinical chemistry mean values the hypotheses of normality and homogeneity of variance were first evaluated, then a Dunn's test, a Dunnett's test or a Mann‐Whitney test was performed, depending on the results of the normality and homogeneity tests (Figure S1; see Supporting Information). The statistical analysis of organ weight data followed a different sequence (Figure S2; see Supporting Information). Unless otherwise stated, all tests were performed two‐sided at level .05.

## 3 RESULTS

### 3.1 Study 1: biodistribution study after a single injection in rats

Rats were injected with 200 μL of control saline or ChAd3‐EBO‐Z at 0.8 × 10^11^ Vp/mL, meaning 1.6 × 10^10^ Vp/animal.

#### 3.1.1 Clinical signs

The vaccine was well tolerated and the only clinical signs were erythema and/or scabs on the right hind limb and at the injection site during the first week after injection; females were generally more affected than males. These findings were considered unrelated to the test item, as they were transient, they occurred across the groups and were observed in controls. It was therefore considered that they were probably due to the clipping and/or the injection procedure. Body weight and food consumption were not affected by the treatment (data not shown).

#### 3.1.2 Biodistribution

The results of ChAd3‐EBO‐Z DNA detection in blood and tissue samples are described in Table 1. No PCR inhibition was observed in any of the samples (data not shown). ChAd3‐EBO‐Z was not detected or quantified in brain, heart, inguinal lymph node, kidney, lung, ovary or testis samples on day 2 or day 8. ChAd3‐EBO‐Z could be quantified in one female liver sample, one male and two female blood samples, two male and one female popliteal lymph nodes, and two male and two female spleen samples (of five per sex) 24 hours after the injection (day 2). On days 8 and 29, ChAd3‐EBO‐Z was undetectable or unquantifiable in these four organs. ChAd3‐EBO‐Z could be quantified in nine of 10 iliac lymph node samples and in all muscle (injection site) samples on days 2, 8 and 29. On day 49, ChAd3‐EBO‐Z was quantified in eight of 10 iliac lymph node samples and in seven of 10 muscle (injection site) samples. Interestingly, ChAd3‐EBO‐Z was not detected in inguinal lymph nodes, at any time point. The ChAd3‐EBO‐Z Vp numbers per μg DNA decreased with time, from day 2 to day 49. The highest ChAd3‐EBO‐Z quantity was detected in the muscle (injection site) at all time points.

**Table 1:** Study 1. Quantification of ChAD3‐EBO‐Z in blood and tissue samples of ChAd3‐EBO‐Z‐treated animals (n = 5 animals per sex)

Tissues	Mean Vp/μg DNA			
	Day 2 (24 h PI)	Day 8 (7 days PI)	Day 29 (28 days PI)	Day 49 (48 days PI)
Blood	3 540 (3)	BLD	BLD	NA
Brain	BLD	BLD	NA	NA
Heart	BLD	BLD	NA	NA
Kidney	BLD	BLD	NA	NA
Liver	418 (1)	BLD	NA	NA
Lung	BLD	BLD	NA	NA
Muscle (injection site)	493 000 (10)	31 900 (10)	16 400 (9)	9 690 (7)
Ovary	BLD	BLD	NA	NA
Iliac lymph node	156 000 (9)	20 400 (9)	14 200 (9)	8 580 (8)
Inguinal lymph node	BLD/Q	BLD	NA	NA
Popliteal lymph node	44 700 (3)	BLD	BLD/Q	NA
Spleen	873 (4)	BLD	BLD	NA
Testis	BLD	BLD	NA	NA

BLD/Q, Below the limit of detection (<25 Vp/well) or quantification (<100 Vp/well); NA, not analyzed; PI, postinjection; (), number of animals included in the mean value (other animals in the group were BLD/Q).

The test item was observed in blood on day 2 in three animals and the presence of ChAd3‐EBO‐Z in tissues such as the spleen or liver on day 2 demonstrated that it was rapidly transported through the blood to these tissues. However, in these organs the test item was already undetectable on day 8 and later.

#### 3.1.3 Pathology

Concerning organ weights, a marked increase was noted as soon as day 2 in the draining inguinal and iliac lymph nodes of the test item treatment animals (up to +672% on day 2 and +435% on day 8, compared with control) and, to a lesser extent, in the popliteal lymph node. Such increased weights correlated with the macroscopic enlargement of the iliac lymph nodes seen in some treated animals at all time points. Minimal increases in the spleen weights (up to +38%) were also noted in both sexes. Lymph nodes and spleen are secondary lymphoid organs, and these organ weight increases were interpreted as the indication that an immunological reaction took place after the administration of ChAd3‐EBO‐Z.

Other organ weight changes were not considered related to ChAd3‐EBO‐Z as they were of low amplitude, were seen at one time point only, and/or were not consistent in males and females. The observed variations in ovary weights were considered related to the different stages of development of corpora lutea that occur during the estrous cycle (Greaves, 2012; Westwood, 2008).

### 3.2 Study 2: repeated‐dose study in rabbits

Rabbits were injected by the IM route with 2 mL of saline (control) or ChAd3‐EBO‐Z at 0.8 × 10^11^ Vp/mL (administered at two injection sites, 1 mL/injection site for a total dose of 1.6 × 10^11^ Vp/animal) on two occasions with a 2‐week interval. Vaccine uptake was assessed by measuring anti‐GP antibodies in serum before treatment and on days 18 and 42. ELISA results showed that an immune response occurred in all treated animals. All had antibody levels high enough to ensure that they had been exposed to the vaccine. The anti‐GP IgG GMT ranged between 224.38 and 1420.71 μg/mL on day 18 and between 1160.90 and 4374.03 μg/mL on day 42.

#### 3.2.1 Clinical signs

No test item treatment‐related clinical signs were observed. Some hematomas at the injection sites were observed transiently in the control and test item groups, and were considered due to the puncture of subcutaneous blood vessels. No ophthalmological findings were observed in any animal. In contrast, changes in rectal temperature could be attributed to the test item as rectal temperature was higher in the animals that received ChAd3‐EBO‐Z than in controls 8 hours (40.2 °C vs. 39.4 °C in males, not statistically significant) and 24 hours after the first injection (40.0 °C vs. 39.0 °C in males [*P* < 0.05] and 40.7 vs. 39.0 in females [*P* <0.01], respectively). Changes in rectal temperature were also observed 8 hours after the second immunization (40.2 °C vs. 39.1 °C in males [*P* < 0.01] and 40.5 vs. 39.4 in females [*P* < 0.01], respectively), but not at later time points.

Body weight loss (ranging from 0.2% to 0.8%) was observed in control animals 24 hours after each injection. In treated animals of both sexes, the body weight loss was greater, ranging from 2.1% to 2.7%. These differences were statistically significant (*P* < 0.01) and were thus attributed to ChAd3‐EBO‐Z. However, this effect was minimal, transient and reversible. Likewise, lower food consumption in test item‐treated animals, when compared with controls, was noted on the 2 days following each injection; this could have been related to the observed body weight loss.

#### 3.2.2 Hematology

**Figure 1:**
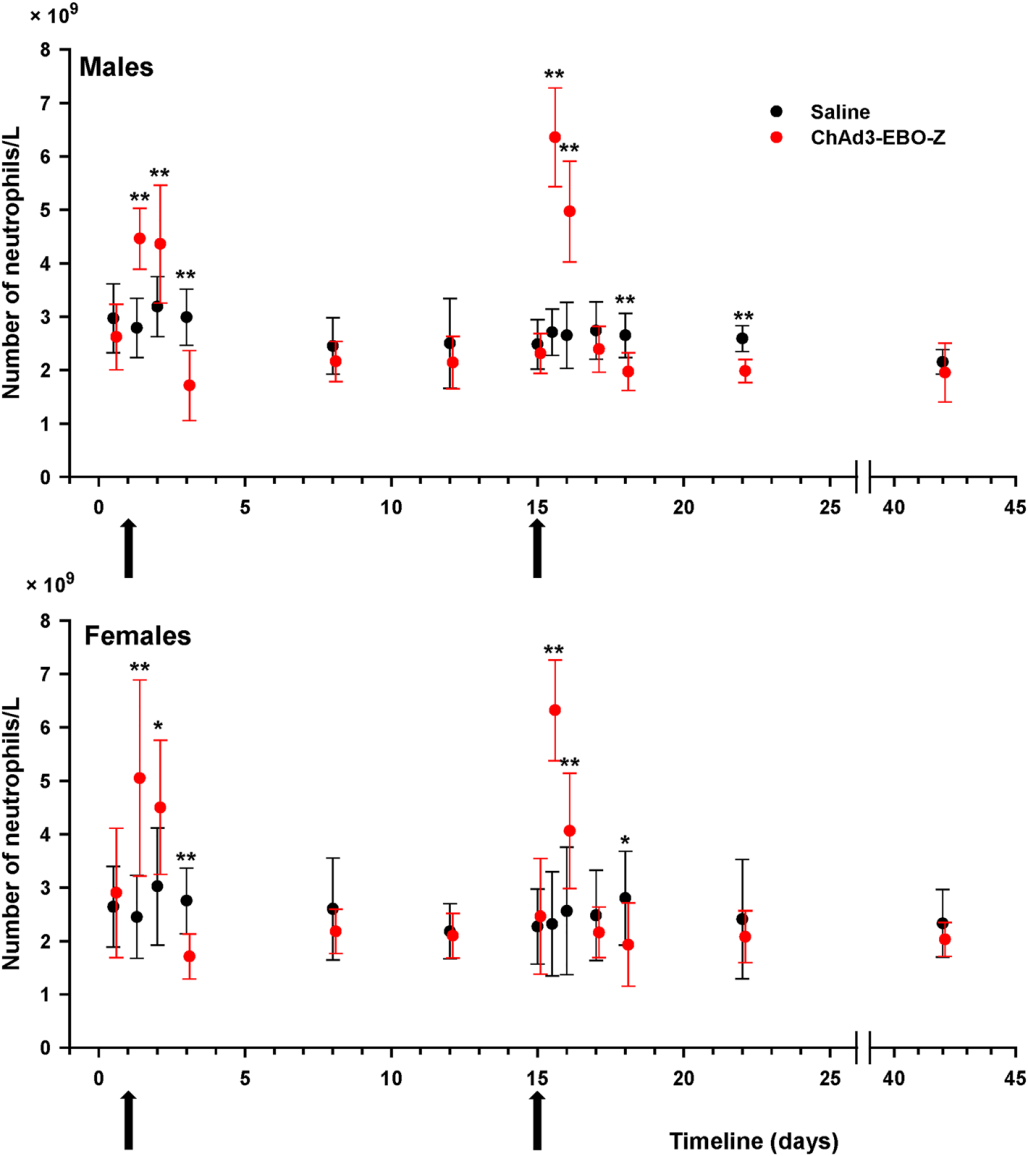
Male and female rabbits were immunized on two occasions (arrows on day 1 and day 15) with either ChAD3‐EBO‐Z or saline. Neutrophil counts were determined at the different displayed time points and expressed as mean ± SD. *P < 0.05 and **P < 0.01, compared with control at each time point [Colour figure can be viewed at wileyonlinelibrary.com]

**Figure 2:**
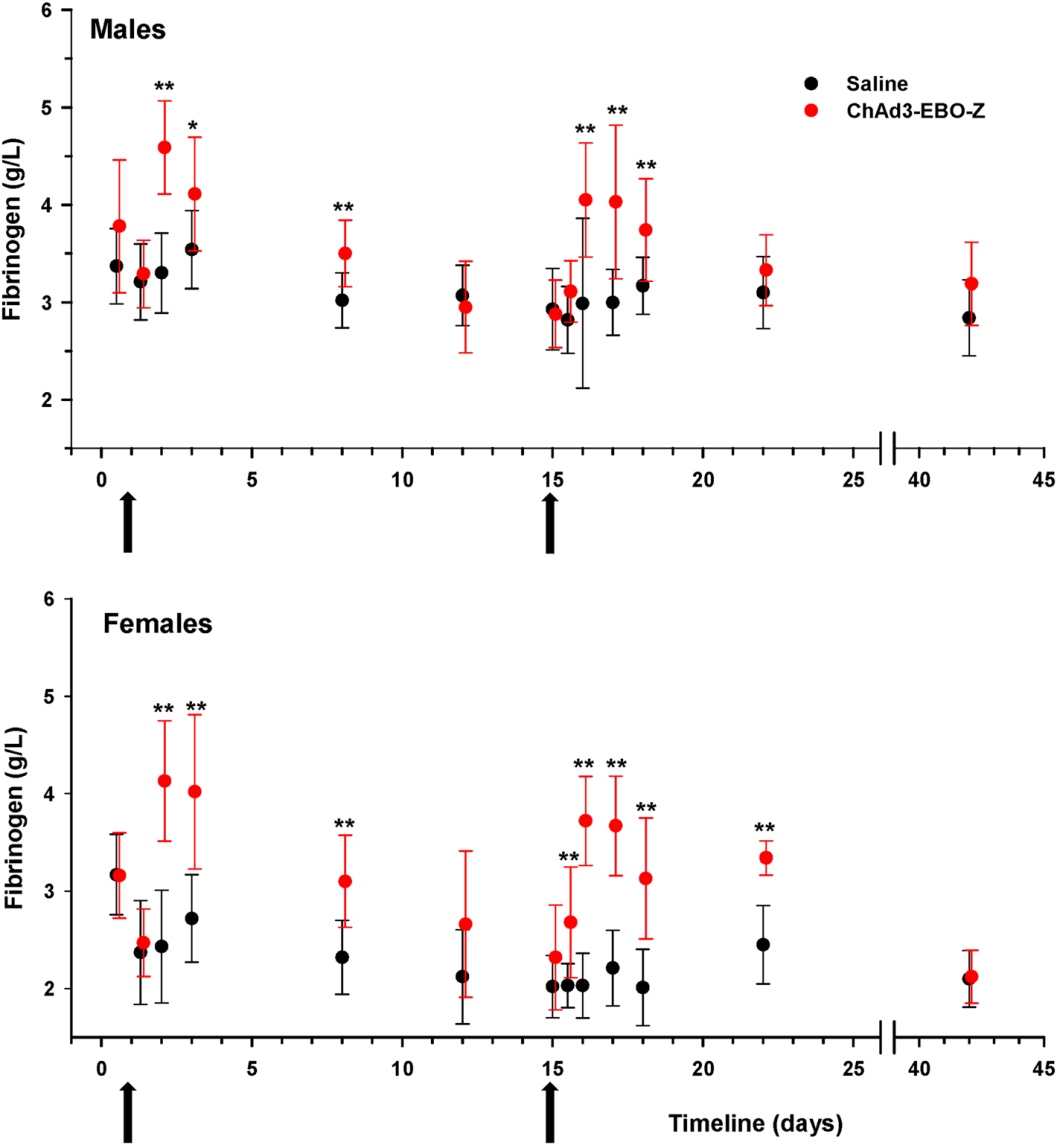
Male and female rabbits were immunized on two occasions (arrows on day 1 and day 15) with either ChAD3‐EBO‐Z or saline. Fibrinogen levels were measured at the different displayed time points and expressed as mean ± SD. *P < 0.05 and **P < 0.01, compared with control at each time point [Colour figure can be viewed at wileyonlinelibrary.com]

Test item‐related effects were observed on some hematology parameters. Notable increased neutrophil count was observed 8 and 24 hours after each injection of ChAd3‐EBO‐Z (Figure 1). This increase was pronounced after the second injection and suggested an inflammatory process in reaction to vaccine administration. The neutrophil increase was followed by a decrease occurring 48 hours after the first immunization and 48 and 72 hours after the second injection. Nevertheless, the variations in neutrophil counts were considered as slight and therefore of no toxicological importance. Likewise, fibrinogen concentration was found to increase considerably after administration of ChAd3‐EBO‐Z in both males and females (Figure 2). This increase started 8 hours after the injection and lasted for up to 7 days. This event could have been related to the inflammation seen microscopically at the injection sites and correlated with the increased neutrophil counts. It demonstrates the establishment of an inflammatory reaction consecutive to vaccine administration. Increased white blood cell counts were also observed 8 hours (statistically significant in females only) or 24 hours after the injections, mainly following the second immunization, which were also considered related to the test item administration.

**Figure 3:**
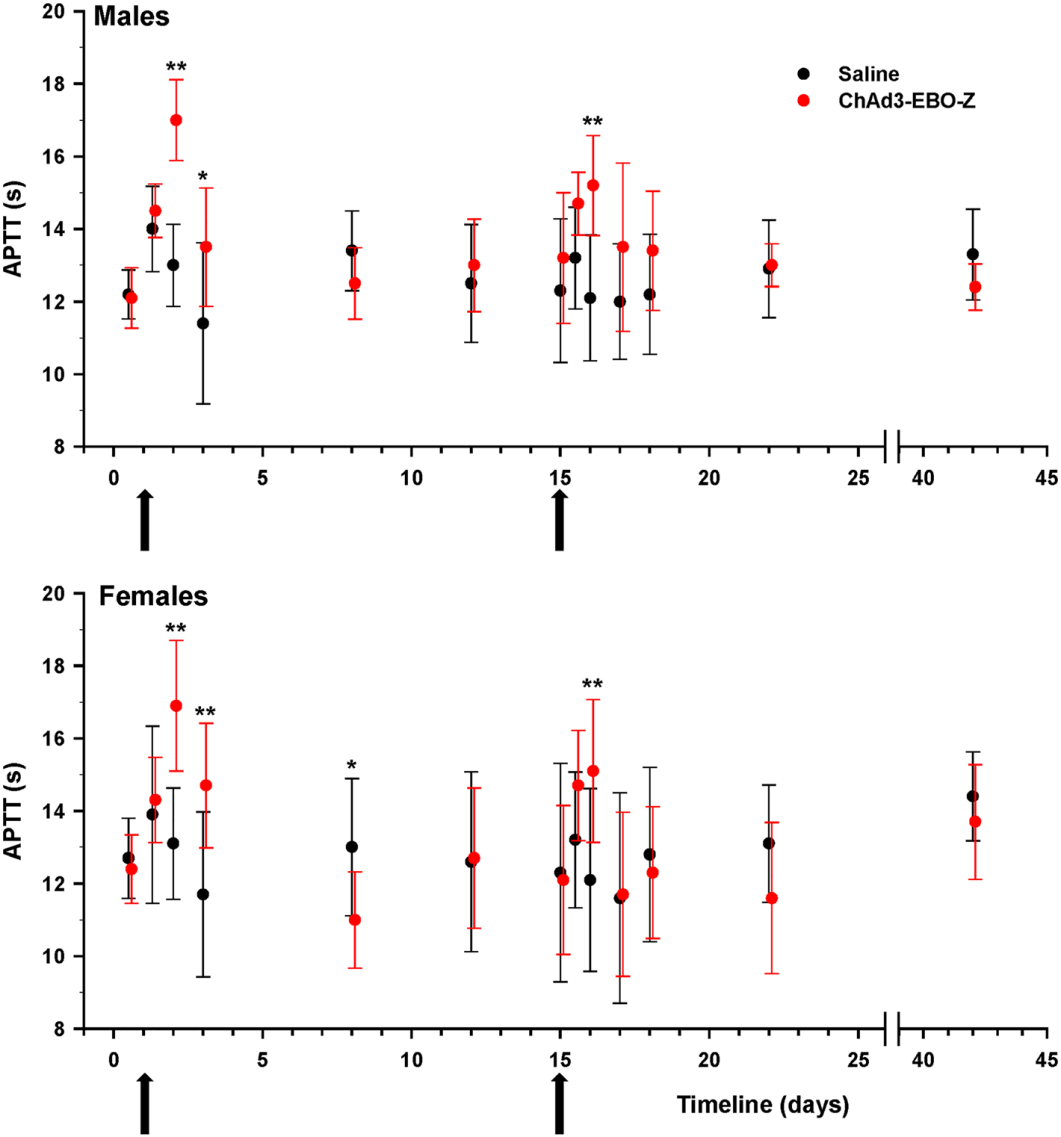
Male and female rabbits were immunized on two occasions (arrows on day 1 and day 15) with either ChAD3‐EBO‐Z or saline. APTT was measured at the different displayed time points and expressed as the number of seconds (mean ± SD). *P < 0.05 and **P < 0.01, compared with control at each time point. APTT, activated partial thromboplastin time [Colour figure can be viewed at wileyonlinelibrary.com]

Statistically significant prolongation of APTT was noted 24 hours after both injections and 48 hours after the first injection in males and females treated with ChAd3‐EBO‐Z candidate vaccine (Figure 3).

Other hematology parameters were decreased following the injection of ChAd3‐EBO‐Z. There was a statistically significant decrease in lymphocyte count 8 hours after each injection and 24 hours after the first injection in males and females (data not shown). This correlated with the increased lymphoid atrophy observed at microscopic examination of the thymus at early necropsy. In addition, decreases (sometimes statistically significant) in eosinophil and monocyte counts were noted 8 and/or 24 hours after ChAd3‐EBO‐Z injection, and these counts were increased (sometimes statistically significant) 48 hours after injection (data not shown). These changes could be indicative of an acute inflammatory reaction or part of a stress leukogram pattern (associated with changes seen in neutrophil counts). The increased monocyte counts noted 48 hours after each injection could reflect a recruitment of macrophages in tissues and it correlated with mononuclear inflammatory cells infiltration seen in the injection sites at microscopic examination.

**Figure 4:**
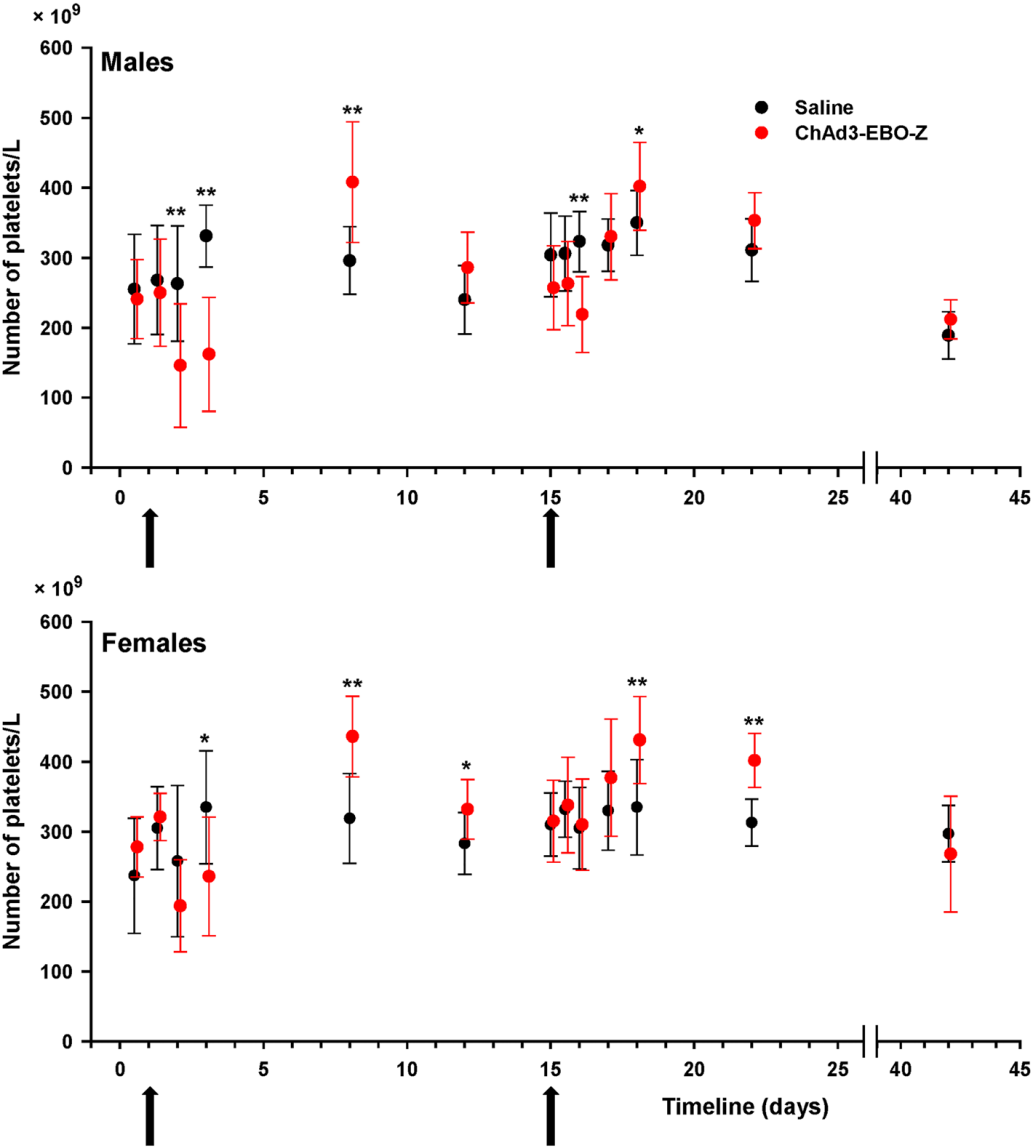
Male and female rabbits were immunized on two occasions (arrows on day 1 and day 15) with either ChAD3‐EBO‐Z or saline. Platelets counts were determined at the different displayed time points and expressed as mean ± SD. *P < 0.05 and **P < 0.01, compared with control at each time point [Colour figure can be viewed at wileyonlinelibrary.com]

Platelet counts were also decreased 24 and 48 hours after the first injection of the test item in both males and females. It was followed by an increase on days 8 and 12. After the second injection, the decrease in platelet count was seen 24 hours later in males only and was less pronounced than after the first injection. It was followed by an increase in the following time points (Figure 4). These changes were not considered adverse, as they were slight, and fully reversible.

Decreased reticulocyte count was noted 48 hours after the first injection in test item‐treated animals of both sexes (*P* < 0.05 for females and *P* < 0.01 for males). These decreases in reticulocytes may be indicative of reduced erythropoiesis and were considered treatment‐related, but not adverse as they were transient and did not induce any changes in red blood cell count. The subsequent increase in reticulocyte count seen from day 8 (still noted on day 15 before the second injection) in control and treated animals was probably due to the repeated blood sampling performed in all animals during the study (higher on day 8 in females treated with the test item).

Finally, starting 24 hours after injection of ChAd3‐EBO‐Z, the large unstained cell count was increased in animals of both sexes, and this lasted for 7 days. These changes were considered due to the test item, but their biological significance remains unclear.

#### 3.2.3 Blood biochemistry

**Figure 5:**
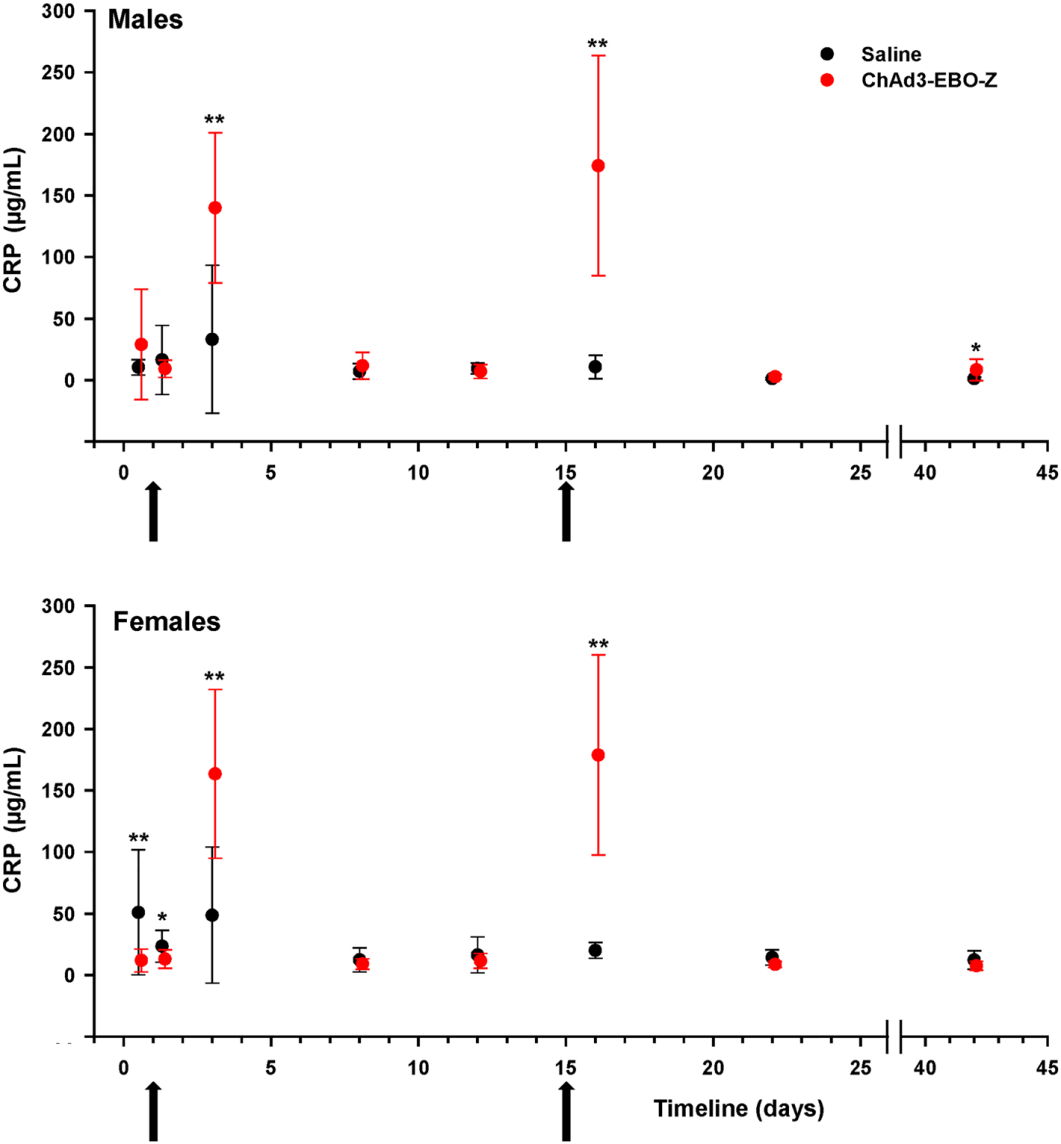
Male and female rabbits were immunized on two occasions (arrows on day 1 and day 15) with either ChAD3‐EBO‐Z or saline. CRP levels were determined at the different displayed time points and expressed as mean ± SD. *P < 0.05 and **P < 0.01, compared with control at each time point. CRP, C‐reactive protein [Colour figure can be viewed at wileyonlinelibrary.com]

Among the clinical chemistry parameters, only CRP levels were shown to be affected consistently by the injections of ChAd3‐EBO‐Z (Figure 5). Two days after the first injection and 1 day after the second injection, CRP levels increased significantly when compared with control values (4.8‐fold in males and 13.6‐fold in females after the first injection; 6‐fold in males and 14.9‐fold in females after the second injection). These increases correlated with the evidence of an inflammatory reaction at hematology (increased neutrophil count and fibrinogen concentration) and at microscopic examination of the injection sites (inflammatory cell infiltrates). CRP levels returned to baseline within one week.

Minimal changes were noted in cholesterol and triglyceride levels at some time points, but a link with the treatment was considered doubtful, as the changes were variable and in opposite directions, or had already been noticed before treatment.

Differences in some enzyme activities (e.g., alkaline phosphatase and creatine kinase) were also noted, but taking into consideration the amplitude and/or direction of the changes they were considered as incidental and not related to treatment. All other changes were considered as incidental and not treatment‐related.

#### 3.2.4 Pathology

On day 18, markedly higher absolute and relative‐to‐body or relative‐to‐brain weights were observed for the iliac lymph nodes in females treated with ChAd3‐EBO‐Z when compared with controls (up to +660%, *P* < 0.01) (Table 2). The inguinal and popliteal lymph nodes showed smaller increases in weight, not reaching statistical significance (Table 2). The increases were also smaller in males for the three analyzed lymph nodes, and did not reach statistical significance (Table 2). These differences correlated with an increased development of germinal centers compared with controls, as observed at microscopic examination. Other variations in lymph node weights (including nondraining mandibular and cervical lymph nodes) were considered of no toxicological importance in view of their low magnitude and the absence of microscopic correlates. Concerning the spleen, slightly higher absolute and relative‐to‐body and relative‐to‐brain weights were observed in females treated with ChAd3‐EBO‐Z (up to +61%; *P* < 0.01) when compared with controls (Table 2). This correlated with the increase in severity and incidence of germinal centers seen microscopically in this organ. No changes were seen in males.

**Table 2:** Study 2. Changes in mean absolute, relative‐to‐body and relative‐to‐brain weights of some organs of the immune system in ChAd3‐EBO‐Z‐treated rabbits (variations in percentage compared with controls)

	Early necropsy (day 18)		Late necropsy (day 43)	
	% change	% change	% change	% change
	Males (n = 5)	Females (n = 5)	Males (n = 5)	Females (n = 5)
**Iliac lymph node**				
Absolute	+113	+641**	+35	+52
Relative‐to‐body	+121	+623**	+29	+50
Relative‐to‐brain	+123	+660**	+24	+35
**Inguinal lymph node**				
Absolute	+70	+46	−1	+36
Relative‐to‐body	+77	+45	−5	+34
Relative‐to‐brain	+78	+50	−10	+22
**Popliteal lymph node**				
Absolute	+19	+28	+29	+10
Relative‐to‐body	+24	+27	+24	+10
Relative‐to‐brain	+23	+32	+17	−1
**Spleen**				
Absolute	−4	+55*	+34	−7
Relative‐to‐body	0	+54**	+29	−7
Relative‐to‐brain	−2	+61**	+22	−16
**Thymus**				
Absolute	−22	−43**	+24	+11
Relative‐to‐body	−19	−44**	+18	+12
Relative‐to‐brain	−20	−41**	+13	−2

* *P* <0.05.

** *P* < 0.01, compared with controls.

In the thymus, moderately lower absolute and relative‐to‐body and relative‐to‐brain weights were observed in males and females treated with ChAd3‐EBO‐Z (up to −44%; *P* < 0.01 or 0.05) when compared with controls. The other organ weight changes were considered as nontest item‐related because they were of insufficient magnitude and/or were uncorrelated with microscopic findings.

On day 43, although not statistically significant, increases in the weights of the lymph nodes draining the injection sites were observed in ChAd3‐EBO‐Z‐treated animals (up to +52% for the iliac lymph nodes in females), compared with controls. The weight of the spleen was also increased up to 34% in this group. These differences were considered related to the test item administration, as they correlated with increases in the severity and incidence of germinal centers seen microscopically in the iliac lymph nodes and spleen.

The other organ weight changes were considered unrelated to the test item because they were of insufficient magnitude and/or had no microscopic correlates.

#### 3.2.5 Macroscopic examinations

On day 18, enlargement of the iliac, inguinal and popliteal lymph nodes and spleen was observed in some males and females treated with ChAd3‐EBO‐Z. This correlated with the increased germinal center development seen at microscopic examination. An increased incidence of red discoloration was observed at injection site 4 and correlated with the increased hemorrhage observed microscopically.

The few other gross observations were considered fortuitous, and corresponded to spontaneous findings encountered in rabbits of this strain and age.

On day 43, no test item‐related macroscopic post‐mortem findings were observed at a systemic level or at injection sites. This suggested total recovery of the formerly observed gross lesions in lymph nodes, spleen and injection site 4. The gross observations were considered consistent with spontaneous findings encountered in rabbits of this strain and age.

#### 3.2.6 Microscopic examinations

On day 18, there were no microscopic test item‐related findings at a systemic level, except for minimal to slight lymphoid atrophy in the thymus of one of five males and three of five females. This correlated with the decrease in thymus weights. A relationship with the test item administration could not be excluded, although this finding was encountered in one control female, which had minimal thymus atrophy on day 43.

Increased severity and/or incidence of germinal center development were noted in the spleen, iliac and popliteal (females only) lymph nodes of animals treated with ChAd3‐EBO‐Z, correlating with increased organ weights and macroscopic enlargement. This was accompanied by increased plasma cell numbers in the iliac lymph nodes in males. No clear microscopic effect was seen in the inguinal lymph nodes. These findings in spleen and lymph nodes draining the injection sites were attributed to the immune response triggered by ChAd3‐EBO‐Z.

Table 3 shows the microscopic observations at injection sites 3 and 4 on day 18, 3 days after the (second) injection. The test item‐related findings consisted of increased incidence and severity of mononuclear inflammatory cell infiltrates (mainly macrophages and lymphoid cells), increased incidence of interstitial hemorrhage in muscle, which correlated with an increased incidence of macroscopic red discoloration that was related to the injection procedure and probably exacerbated by vaccine administration. Interstitial eosinophilic fibrillar material (suggestive of fibrin) was also observed, and rare degeneration/necrosis of myofibers was seen with higher severity/incidence in males and/or females treated with ChAd3‐EBO‐Z when compared with saline controls. At the injection sites in controls, occasional infiltrates of mononuclear cells, hemorrhage and rare degeneration/necrosis of myofibers were observed and were considered related to the injection procedure and/or saline administration.

**Table 3:** Study 2. Incidence and severity of the injection site‐related microscopic findings on day 18 (3 days after the second injection for sites 3 and 4 and 17 days after the first injection for sites 1 and 2)

Sex	Males		Females	
Group	Saline	ChAd3‐EBO‐Z	Saline	ChAd3‐EBO‐Z
Number of examined animals	5	5	5	5
**Injection site 1**				
Infiltrate mononuclear inflammatory cells				
Grade 1	3	3	1	4
Grade 2	0	1	0	0
Hemorrhage				
Grade 2	0	0	0	1
Eosinophilic material				
Grade 1	0	1	0	0
Mineralization				
Grade 1	0	1	0	0
Fibroplasia/fibrosis				
Grade 1	0	0	0	2
**Injection site 2**				
Infiltrate mononuclear inflammatory cells				
Grade 1	1	3	0	5
Grade 2	0	1	0	0
Hemorrhage				
Grade 1	0	1	0	0
Grade 2	0	1	0	0
Eosinophilic material				
Grade 1	0	2	0	0
Grade 2	0	0	0	1
Mineralization				
Grade 1	0	2	0	0
Fibroplasia/fibrosis				
Grade 1	0	2	0	1
**Injection site 3**				
Infiltrate mononuclear inflammatory cells				
Grade 1	1	3	0	0
Grade 2	0	1	1	0
Grade 3	0	0	0	1
Hemorrhage				
Grade 1	1	1	1	0
Grade 2	0	0	0	1
Eosinophilic material				
Grade 1	0	1	0	1
**Injection site 4**				
Infiltrate mononuclear inflammatory cells				
Grade 1	0	3	2	2
Grade 2	0	2	1	3
Hemorrhage				
Grade 1	0	4	1	2
Grade 2	0	0	0	2
Eosinophilic material				
Grade 1	0	3	0	2

The microscopic observations at injection sites 1 and 2 on day 18, i.e., 17 days after the first injection of ChAd3‐EBO‐Z, consisted of an increased incidence and severity of mononuclear cell infiltrates (mainly macrophages and lymphoid cells), and an increased incidence of occasional interstitial hemorrhage in the muscle (injection site) of males and females. This was considered related to the injection procedure and apparently exacerbated by vaccine administration. There was occasional interstitial eosinophilic fibrillar material (suggestive of fibrin), mineralization in some males, and the presence of fibroplasia/fibrosis. The fibroplasia/fibrosis that was seen in sites 1 and 2 and not in sites 3 and 4 on day 18 in test item‐treated animals was consistent with the chronology of the injections, and suggested the resolution of the remaining inflammation elicited by vaccine injection. In sites from controls, occasional minimal infiltrates of mononuclear cells were observed and considered related to the previous injection procedure and/or saline administration.

The other microscopic findings were not considered associated with the test item as they were consistent with spontaneous background findings found in controls and described in the literature (McInnes, 2012) and, generally, the findings were distributed randomly among the groups.

On day 43, there were no microscopic ChAd3‐EBO‐Z‐related findings at the systemic level. Increased severity and/or incidence of germinal center development was noted in the iliac lymph nodes and spleen of ChAd3‐EBO‐Z‐treated animals, but the magnitude of these changes remained at a level similar to that observed at the early necropsy. Concerning the injection sites, only minimal infiltrates of mononuclear inflammatory cells were observed, with a severity level similar to that of controls, but with a higher incidence in the test item‐treated group.

The other microscopic findings were not considered related to the test item because these findings were consistent with spontaneous background findings described in the controls and in the literature (McInnes, 2012), and they were generally randomly distributed among the groups. They included incidental congenital, degenerative and inflammatory changes of low grade.

In conclusion, the test item was considered locally well tolerated and the inflammatory lesions had regressed well on day 43, i.e., 28 days after the last injection, thus suggesting almost complete reversibility.

## 4 DISCUSSION

The ChAd3‐EBO‐Z candidate vaccine was evaluated for its biodistribution after a single IM injection in rats, and was further evaluated in a repeated‐dose toxicity study after two IM administrations at a 2‐week interval in rabbits.

Although the adenoviral vector used is replication‐deficient, entailing that no de novo functional viral particles can be generated, it may disseminate outside the injection site and the potential targets should be identified. After a single IM administration of the candidate vaccine, ChAd3‐EBO‐Z DNA was mainly found in the muscle at the injection site and in the draining iliac lymph nodes 1 day later, reflecting the capture of the test item by the immune system of the host. The amount of viral DNA decreased over time in these organs up to 48 days after treatment, indicating clearance. A transient exposure to ChAd3‐EBO‐Z was seen in the blood, liver, popliteal lymph node and spleen, but ChAd3‐EBO‐Z was not detected in brain, heart, inguinal lymph node, kidney, lung, ovary and testes. Overall, these observations are in line with previous ones made with human adenovirus type 5‐ and type 35‐vectored vaccines given IM to rabbits (Sheets et al., 2008). The persistence of adenoviral DNA, as measured by qPCR, for a few months after IM injection at the injection site and in draining lymph nodes has been consistently observed in our (unpublished data) and other (Sheets et al., 2008) studies with a variety of adenoviral vectors. The translatability of these results to humans and the potential impact on safety is not known. Of note, biodistribution data are based on the measurement of adenoviral DNA, which cannot distinguish between functional (infective) viral particles or noninfective/degraded adenoviruses. One should also consider that the administered dose in the biodistribution study was at least 20‐fold the human dose relative to body weight. Finally, the quantities noted in the sampled tissues/organs at the final time point (day 49) were <30 000 copies/μg host cell DNA, which is below the threshold level for which further studies would be required (FDA‐CBER, 2007; WHO, 2005).

In this biodistribution study, the single administration of ChAd3‐EBO‐Z was not associated with changes in general clinical signs, and the macroscopic or organ weights changes reported for the draining lymph nodes and spleen reflected the expected inflammatory mechanisms that occur as part of the immune response triggered by a vaccine.

Inflammatory reactions, indicating the establishment of an immune response, were also observed after each of the two injections in the repeated‐dose study in rabbits. Particularly, two hematology parameters (neutrophil counts and fibrinogen concentration) and one clinical chemistry parameter (CRP level), were shown to be transiently affected after each ChAd3‐EBO‐Z administration. The CRP level increase is claimed as a good indicator of vaccine‐induced cytotoxic T‐cell activation or T‐helper 1 type immune response involvement (Destexhe et al., 2013; Green, 2015). Changes indicative of the establishment of an immune response were also demonstrated by the activated appearance of lymphoid organs (such as draining lymph nodes and spleen) at macroscopic and microscopic examination. These results are in line with general observations consecutive to vaccine administration in the frame of toxicity studies (Baldrick, 2016).

More specific to our study, minimal prolongation of APTT was seen 24 hours after both injections and 48 hours after the first injection in animals of both sexes. Similar APTT prolongation has already been observed in a rabbit study after IM injection of adenovirus‐vectored vaccines (Sheets et al., 2008) and clinically upon adenovirus infection or after adenovirus vector delivery (Beck, Strauss, Kisker, & Henriksen, 1979; Jaeger et al., 1993; Malaeb et al., 2005). However, the prolongation of APTT in those clinical studies seemed artifactual, and said to be due to the presence of inflammation‐induced antiphospholipid antibodies in the serum of infected or inoculated individuals, which impacted the assay. Whether this also applies to rabbits is uncertain and has not been investigated further. Nevertheless, it should be noted that this phenomenon was transient and APTT returned to normal within a few days.

Furthermore, we observed that some hematology parameters decreased upon administration of ChAd3‐EBO‐Z, notably the platelet counts. Thrombocytopenia is a commonly reported symptom during viral infections. Platelets can act as an antimicrobial defense system and interact with multiple viral pathogens in various ways, one way being the binding and subsequent sequestration by the reticuloendothelial cells (Assinger, 2014). More specifically, adenovirus‐induced thrombocytopenia is a known phenomenon, as it has already been described in different animal models (Cichon et al., 1999; Othman, Labelle, Mazzetti, Elbatarny, & Lillicrap, 2007; Varnavski, Calcedo, Bove, Gao, & Wilson, 2005; Wolins et al., 2003) and in humans (Raper et al., 2002) after parenteral administration. A suggested mechanism is that adenovirus binding to platelets is followed by their sequestration and degradation in the liver, which has been described after intravenous administration of adenovirus 5 (Ad5)‐based vectors. Indeed, in mice, Ad5‐based vectors were found to co‐localize with platelets and liver Kupffer cells/macrophages (Stone et al., 2007). Furthermore, the platelet receptor GPIIb/IIIa has been suggested to be involved in the binding (Assinger, 2014), and factors such as van Willebrand factor may facilitate the interaction (Othman et al., 2007).

In our study, platelet count decreases were modest and transient, with no clinical signs or histopathological correlates, which may relate to the fact that the adenovirus‐mediated thrombocytopenia is a dose‐dependent effect (Cichon et al., 1999; Wolins et al., 2003). Indeed, it is the number of virus particles circulating in the blood that determines the magnitude of the platelet count decrease. After intravenous administration, as is the case in all works cited in the previous paragraph, the concentration of adenovirus in the blood is maximal and consequently the effect is pronounced. After IM administration, as is the case with our vaccine, the diffusion of adenovirus vectors into the blood is expected to be marginal, leading to a milder effect on platelets. In this regard, our results are in line with earlier observations made in rabbit after IM administration of adenovirus vectors (Sheets et al., 2008). Overall, the platelet count changes observed in our study were considered transient pathophysiological responses to vaccine administration that were not associated with any apparent adverse consequence.

In conclusion, under the conditions of these studies, single and repeated administration of the ChAd3‐EBO‐Z candidate vaccine was locally and systemically well‐tolerated, with only transient signs of inflammatory reactions and a modest drop in platelet count. Based on these results, the ChAd3‐EBO‐Z candidate vaccine is considered suitable for human clinical trials in healthy volunteers. The clinical phase of this vaccine, which was advanced in response to a request from the WHO with respect to the Ebola outbreak in West Africa, demonstrated immunogenicity and an acceptable safety profile (De Santis et al., 2016; Tapia et al., 2016). In addition, as the biodistribution and toxicology profiles appear more dependent on the adenoviral platform than on the gene insert itself (Sheets et al., 2008), we suggest that our results can be extended to other ChAd3‐based candidate vaccines.

## FUNDING

This work was sponsored by GlaxoSmithKline Biologicals SA, which was involved in all stages of the study conduct and analysis and took responsibility for all costs incurred in publishing.

## CONFLICT OF INTEREST

C.P., A.M.S. and E.D. are, or were at the time of the study, employees of the GSK group of companies. E.D. and A.M.S. report ownership of GSK shares and/or restricted shares. A.M.S. is listed as inventor on patents owned by GSK. G.C., M.E.D., C.C., C.T.D. and C.S. are employees of Citoxlab, a Contract Research Organization contracted by GSK in the context of this study.

## AUTHOR CONTRIBUTIONS

A.M.S., C.C., C.P., E.D., G.C. and M.E.D. were involved in the conception and design of the study and/or the development of the study protocol. C.C., C.P., C.S., C.T.D. and M.E.D. participated to the acquisition of data. C.C., C.P., C.S., C.T.D., E.D., G.C. and M.E.D. analyzed and interpreted the results. All authors were involved in drafting the manuscript or revising it critically for important intellectual content. All authors had full access to the data and approved the manuscript before it was submitted by the corresponding author.

## Supporting information


**Figure S1.** Decision tree for the statistical analysis of body weight, food consumption, rectal temperature, hematology, blood biochemistry and CRP measurement data.
**Figure S2**. Decision tree for the statistical analysis of organ weight data.
