# Supplementary material for: Nonclinical safety assessment of repeated administration and biodistribution of ChAd3‐EBO‐Z Ebola candidate vaccine
Source: J Appl Toxicol. 2020 Jan 21;40(6):748–62. doi: 10.1002/jat.3941 (PMC7318182; doi:10.1002/jat.3941)
Supplement: Supplementary file 1 — Figure S1. Decision tree for the statistical analysis of body weight, food consumption, rectal temperature, hematology, blood biochemistry and CRP measurement data. Figure S2. Decision tree for the statistical analysis of organ weight data. [file JAT-40-748-s001.docx]

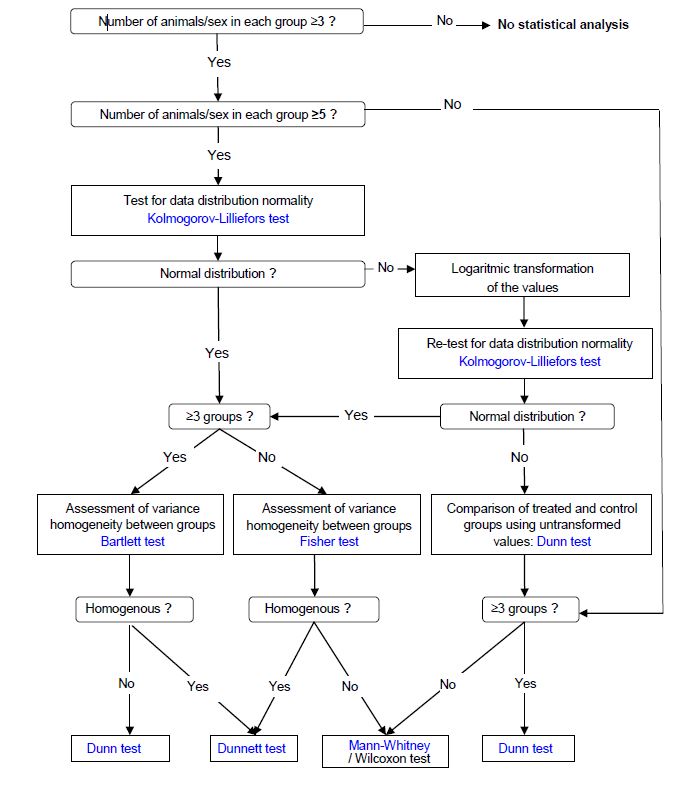


**Supplementary Figure 1.** Decision tree for the statistical analysis of body weight, food consumption, rectal temperature, hematology, blood biochemistry and CRP measurement data.


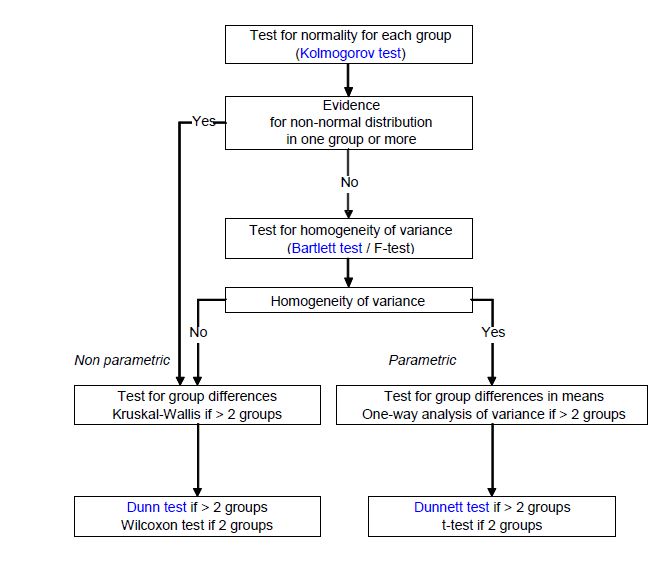


Supplementary Figure 2. Decision tree for the statistical analysis of organ weight data.
